# Cognitive reshaping and resurgence of humanness: restructuring the medical education continuum in the era of generative AI

**DOI:** 10.3389/fmed.2026.1867722

**Published:** 2026-09-11

**Authors:** Ya Liu, Lutuo Han, Linlin Che, Wei Dong, Ting Zhang, Hongwei Guo

**Affiliations:** Heilongjiang University of Chinese Medicine, Harbin, China

**Keywords:** clinical reasoning, competency-based medical education, generative AI, humanness, medical education

## Abstract

**Background:**

Generative artificial intelligence (AI) is rapidly transforming medical education. While AI enhances personalized learning, its integration raises concerns regarding “cognitive outsourcing,” automation bias, and erosion of independent clinical reasoning.

**Aim & methods:**

This narrative review critically evaluates the cognitive and educational impacts of generative AI and proposes structural realignment across the medical education continuum. We synthesized literature from major databases (PubMed, Scopus) focusing on AI's intersection with clinical reasoning and humanistic competencies.

**Main findings:**

We identify three cognitive vulnerabilities: deskilling (loss of diagnostic abilities among advanced learners), never-skilling (failure to develop foundational mental models in junior trainees), and automation bias (uncritical acceptance of machine-generated recommendations). Traditional memory-based assessments, such as multiple-choice questions (MCQs), are increasingly insufficient for evaluating clinical readiness.

**Key recommendations:**

Medical education needs to pivot towards methodologies AI cannot replicate, acknowledging that strategies like case-based learning (CBL) and problem-based learning (PBL) are not novel but are now urgently necessitated by AI. We propose a division of labor where “AI treats the chart” while “humans treat the patient.” Undergraduate medical education (UME) is encouraged to prioritize pathophysiological reasoning to counter never-skilling; graduate medical education (GME) would benefit from emphasizing human-AI collaboration and deliberate reflection to counter deskilling and automation bias; continuing medical education (CME) may consider facilitating lifelong adaptation and structured “unlearning” of obsolete heuristics.

**Conclusion:**

Rather than competing with algorithmic memory, medical education needs to cultivate “augmented clinicians” equipped with high AI literacy and profound humanness, ensuring technology enhances rather than displaces relational patient care.

## 1 Introduction

In recent decades, medical education has successfully transitioned from unidirectional knowledge transmission to competency- and PBL/CBL frameworks. However, the advent of generative AI acts as a powerful catalyst, presenting unprecedented challenges and opportunities to these existing models. Rather than necessitating entirely new educational paradigms, AI drastically lowers the barrier to knowledge acquisition, thereby accelerating the need to emphasize higher-order clinical reasoning and human-like care. Within the framework of precision education, AI-driven adaptive platforms dynamically adjust the difficulty and sequence of clinical cases based on individual learner performance. Early data from a 2025 randomized controlled trial (RCT) suggests that medical students using an AI-driven personalized learning platform showed significant improvements in academic performance (84.47 vs. 81.72, *p* = 0.034), a 41.5% increase in daily learning time, and a 48.3% increase in literature reading volume (1). Furthermore, through AI-driven virtual tutoring and interactive multimodal scenarios, medical education aims to address Bloom's two-sigma problem by providing scalable, individualized feedback comparable to expert one-on-one tutoring, thereby mitigating the cognitive load associated with the expanding volume of medical knowledge (2–4).

The increasing capacity of AI to process medical knowledge necessitates a reevaluation of the core objectives of medical education. While humanness and clinical empathy have always been fundamental to medical practice, the advent of AI re-emphasizes their irreplaceability (5–8). Consequently, medical education is encouraged to facilitate a shift in professional identity, transitioning the physician's role from a primary knowledge repository to a facilitator of patient-centered care and shared decision-making (6). Addressing the tension between efficiency-driven technological tools and human-like care requires the development of collaborative intelligence (9, 10). Under this framework, educational priorities need to increasingly emphasize communication skills, ethical judgment, and narrative medicine.

However, current literature largely addresses AI integration in isolated educational settings without providing a coherent, phase-specific framework that spans the entire training continuum. Moreover, while the risks of over-reliance on AI are frequently mentioned, the critical distinction between beneficial cognitive offloading and detrimental cognitive outsourcing—and their distinct educational consequences—remains insufficiently operationalized for curriculum designers.

Therefore, the aim of this narrative review is twofold: first, to critically examine the three specific cognitive vulnerabilities that generative AI introduces into clinical training—namely, deskilling, never-skilling, and automation bias; and second, to propose a structural realignment across the medical education continuum (Undergraduate, Graduate, and Continuing Medical Education) with phase-specific priorities that directly counter these vulnerabilities. The remainder of this article is structured as follows: Section 2 outlines the methodology of our literature synthesis; Section 3 analyzes the cognitive impacts, distinguishing between beneficial cognitive offloading and detrimental outsourcing; Section 4 proposes phase-specific educational adaptations across UME, GME, and CME; Section 5 discusses the re-emphasis on “humanness” and implementation challenges; and Section 6 concludes with future perspectives.

## 2 Methods

To construct this narrative review, a comprehensive literature search was conducted across major electronic databases, including PubMed, Scopus, and Web of Science, from their inception up to April 2026. The search strategy utilized combinations of the following key terms: (“Generative AI” OR “Artificial Intelligence” OR “Large Language Models”) AND (“Medical Education” OR “Undergraduate Medical Education” OR “Graduate Medical Education” OR “Continuing Medical Education”) AND (“Clinical Reasoning” OR “Humanism” OR “Competency-Based Medical Education”).

Articles were included if they were peer-reviewed, published in English, and specifically addressed the impact, integration, or challenges of generative AI within the medical education continuum. Perspectives, commentaries, and original research articles were all considered to capture a holistic view of this rapidly evolving field. Two authors (YL and LH) independently screened the titles and abstracts for relevance against the inclusion criteria. Full texts of potentially pertinent articles were retrieved and assessed for final inclusion; disagreements during the screening process were resolved through discussion and consensus, with consultation from a third author (HG) when necessary. Reference lists of included articles were also hand-searched to identify additional relevant publications.

As a narrative and critical review rather than a systematic review or meta-analysis, this methodology does not aim to provide a quantitative summary of effect sizes. Instead, it is designed to synthesize current conceptual frameworks, identify key educational vulnerabilities, and propose strategic adaptations based on the available evidence.

Regarding the study selection process, our initial database search yielded approximately 450 potential records. After removing duplicates and screening titles and abstracts for relevance to medical education and clinical reasoning, approximately 120 full-text articles were assessed for eligibility. Ultimately, 69 highly relevant publications were included in the final narrative synthesis to construct the proposed framework.

## 3 The cognitive impact: educational vulnerabilities and the alteration of clinical reasoning

### 3.1 Cognitive offloading vs. cognitive outsourcing

**Figure 1:**
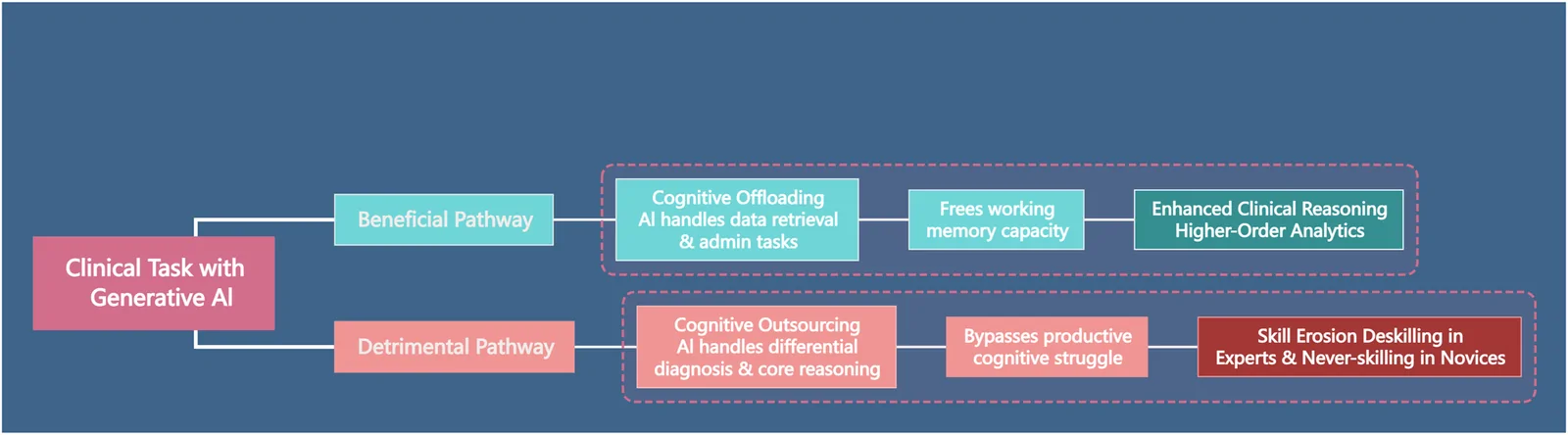
Pathways of AI reliance: cognitive offloading vs. Cognitive Outsourcing. This flowchart conceptualizes the divergent educational outcomes of utilizing generative AI in clinical tasks. The beneficial pathway (top) illustrates cognitive offloading, where delegating administrative and retrieval tasks to AI frees working memory capacity, thereby enhancing higher-order clinical reasoning. Conversely, the detrimental pathway (bottom) illustrates cognitive outsourcing, where delegating core diagnostic reasoning to the algorithm bypasses necessary cognitive struggle, leading to deskilling among experts and never-skilling among novices.

While concepts such as deskilling and automation bias have been well-established in cognitive psychology and aviation literature, this review synthesizes and reconceptualizes these phenomena alongside the specific educational risk of never-skilling. Together, they form a novel theoretical framework to understand the divergent educational outcomes of generative AI. The integration of generative AI into medical curricula necessitates a distinction between cognitive offloading and cognitive outsourcing, as these mechanisms yield divergent educational outcomes. Cognitive offloading refers to the delegation of rote data retrieval or administrative tasks to AI systems. This strategy reduces working memory load, thereby preserving cognitive capacity for higher-order analytic processes and complex clinical reasoning (11). In contrast, cognitive outsourcing involves the delegation of the clinical reasoning process itself to the algorithm. This results in cognitive substitution, wherein AI-generated outputs replace independent trainee analysis (12). The divergence between beneficial cognitive support and detrimental skill erosion can be conceptualized through these two distinct pathways of AI reliance (Figure 1).

Empirical assessments indicate a negative correlation between high-frequency AI reliance and critical thinking metrics, particularly among early-career medical trainees (13, 14). The literature identifies two primary mechanisms of skill degradation associated with this reliance. The first, deskilling, affects advanced learners and practicing clinicians who experience a deterioration of previously acquired diagnostic abilities due to sustained algorithmic dependence (11). The second, never-skilling, occurs when junior trainees are exposed to AI systems prior to establishing foundational clinical mental models. By circumventing the cognitive effort required for initial skill acquisition, these trainees fail to develop essential problem-solving frameworks (15). For example, a junior medical student relying entirely on an LLM to generate a differential diagnosis for acute dyspnea may bypass the internal cognitive modeling required to distinguish between cardiac and pulmonary etiologies. This bypass prevents the formation of foundational illness scripts.

These educational vulnerabilities are compounded by the operational limitations of LLMs. Benchmarking analyses demonstrate that LLMs rely primarily on statistical pattern-matching rather than pathophysiological reasoning (16). In complex differential diagnosis generation, LLMs frequently exhibit inflexible reasoning, demonstrating a tendency toward premature closure rather than maintaining diagnostic uncertainty (15, 16). Furthermore, LLMs assign high confidence intervals to their outputs regardless of clinical accuracy, increasing the probability of reasoning degradation among trainees who adopt these outputs without critical verification.

### 3.2 Automation bias, governance, and liability issues

The uncritical acceptance of machine-generated recommendations, defined as automation bias, introduces measurable risks to diagnostic accuracy. Automation bias operates bidirectionally: it increases reliance on correct AI recommendations while simultaneously diminishing the clinician's ability to identify and reject incorrect outputs (17, 18). A simulated diagnostic study involving 223 healthcare professionals demonstrated that while correct AI support increased the probability of accurate decision-making by a factor of ten, exposure to incorrect AI recommendations reduced diagnostic accuracy significantly below baseline performance (17).

Specific demographic and contextual factors mediate this susceptibility. Non-specialists and junior trainees demonstrate higher rates of false agreement with incorrect AI outputs (18). Furthermore, automation bias positively correlates with verification complexity; as the difficulty of independently corroborating an AI output increases, users are more likely to defer to the algorithm (19). Recent data indicate that the implementation of explainable AI—providing visual or textual rationales for algorithmic decisions—does not consistently mitigate automation bias and can occasionally increase user trust in incorrect recommendations (20).

The use of probabilistic algorithms also presents specific governance and liability challenges in medical education and practice. LLMs are documented to generate confabulations—factually inaccurate medical content presented with high linguistic fluency (21). When trainees externalize clinical judgment to these systems, it obscures the allocation of medical accountability and compromises cognitive autonomy (22, 23). Consequently, deploying LLMs without structured clinical supervision challenges the educational mandate to produce independent practitioners capable of safeguarding patient welfare (22, 23).

### 3.3 The limitations of traditional assessments and the knowledge-practice gap

While the high proficiency of LLMs on standardized examinations does not invalidate MCQs as a reliable measure of human knowledge under strictly proctored conditions, it does necessitate a reevaluation of what we assess. As AI can instantly retrieve factual information, the relative weight of assessing pure rote memory is expected to decrease, prioritizing the evaluation of higher-order application. It is important to note that MCQs remain highly valuable for assessing foundational medical knowledge when appropriately designed. Rather than suggesting that traditional assessments are obsolete, this review emphasizes that assessment programs will likely require complementary methods capable of evaluating higher-order reasoning and human-AI collaboration. Furthermore, traditional open-book assessments, such as case reports or reflective writing assignments, are now highly vulnerable to uncredited AI assistance since their completion cannot be strictly controlled. Systematic reviews document a quantifiable knowledge-practice gap: advanced LLMs achieve 84% to 93% accuracy on standardized medical licensing examinations (e.g., USMLE) but perform at 45% to 69% on practice-based clinical tasks (24, 25). This discrepancy suggests that MCQ performance no longer serves as a reliable proxy for clinical readiness in the context of AI availability.

Experimental studies indicate that LLMs exploit format-specific vulnerabilities within MCQs rather than demonstrating medical comprehension. In controlled settings, LLM performance deteriorated by an average of 39.43% when selected-response items were converted to free-response formats (26). Even under conditions where the question stem was entirely masked, LLMs identified correct answers at rates significantly above random chance by detecting statistical patterns among the options (26). This mechanism parallels human test-taking behaviors such as the cueing effect, which elicits the process of elimination rather than the open-ended clinical reasoning required in authentic patient care (27–29).

The documented limitations of written assessments support a transition toward Competency-Based Medical Education (CBME). To evaluate skills that algorithms cannot currently replicate, curricula are increasingly utilizing Entrustable Professional Activities (EPAs) and workplace-based assessments, prioritizing the direct observation of clinical behaviors (30, 31). Additionally, Script Concordance Testing (SCT) is utilized to measure adaptive clinical judgment under conditions of uncertainty—domains where LLM pattern-matching is inadequate (32, 33). Future assessment frameworks are therefore encouraged to target collaborative intelligence, measuring a trainee's capacity to critically appraise AI outputs, integrate information ethically, and exercise independent clinical judgment (34). Table 1 summarizes this necessary paradigm shift from traditional, memory-based evaluation methods to competency-based assessments designed for the AI era.

**Table 1:** Paradigm shift in medical knowledge assessment in the era of generative AI.

Assessment dimension	Traditional Assessments (Pre-AI Paradigm)	Competency-Based Assessments (AI-Era Paradigm)
Primary Objective	Rote knowledge recall and unidirectional information synthesis	Adaptive clinical judgment, ethical application, and collaborative intelligence
Core Modalities	Multiple-choice questions and standardized written examinations	Workplace-based assessments, Entrustable Professional Activities, Script Concordance Testing
Target Reasoning Skill	Diagnostic reasoning (categorization; focusing on “What is wrong?”)	Management reasoning (prioritization; focusing on “What should we do?”)
Vulnerability to AI	High; LLMs actively exploit cueing effects and statistical patterns within predefined options	Low; requires real-time humanness integration, moral subjectivity, and embodied clinical presence
Verification Mechanism	Algorithmic grading of static, single-best-answer scenarios	Human-in-the-loop (HITL) oversight and dynamic critical appraisal of probabilistic outputs

## 4 Restructuring the medical education continuum

### 4.1 Undergraduate medical education: rebuilding the cognitive foundation

**Figure 2:**
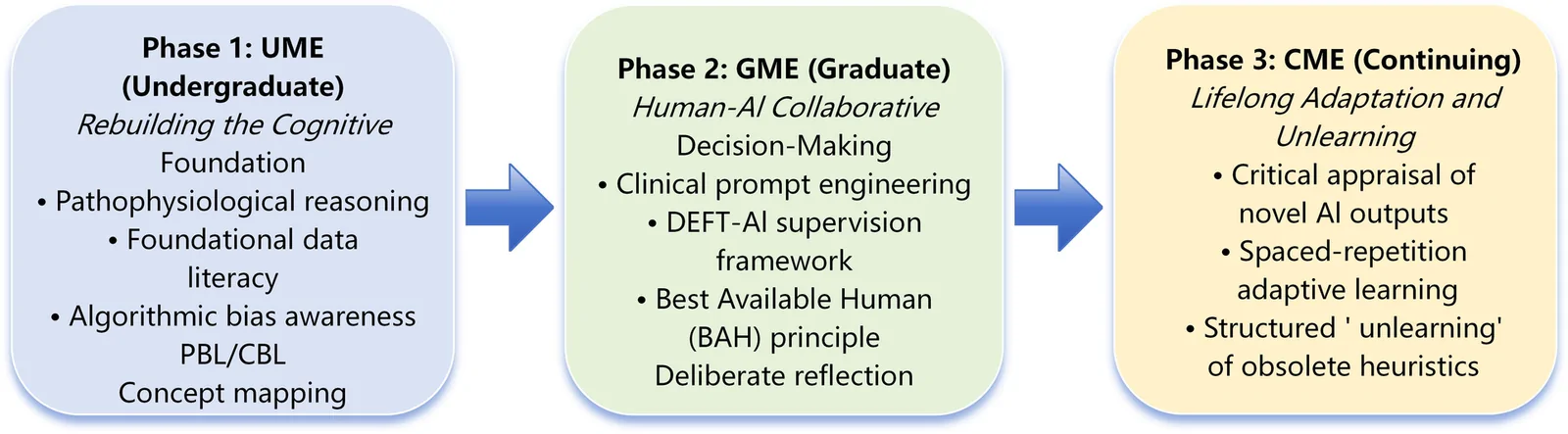
Restructuring the medical education Continuum. A proposed developmental framework mapping phase-specific AI and relational competencies across the medical education continuum. The progression illustrates a paradigm shift from rebuilding foundational mechanistic knowledge and data literacy through active learning strategies (e.g., PBL with AI-generated distractors, concept mapping) in UME—directly countering the never-skilling risk—to mastering human-AI collaborative decision-making and deliberate reflection in GME—countering deskilling and automation bias—and ultimately committing to lifelong adaptation and the structured “unlearning” of obsolete heuristics in CME. Each phase includes associated assessment strategies (oral case presentations, supervised AI-use assessments, and reflective portfolios, respectively).

The integration of generative AI necessitates a structural realignment across the entire medical education continuum (Figure 2). Within UME, a primary concern is the risk of never-skilling—whereby junior trainees are exposed to AI-generated clinical outputs before establishing foundational mental models, thereby bypassing the cognitive effort essential for initial skill acquisition (as discussed in Section 3.1). To counter this vulnerability, UME is encouraged to shift its emphasis from rote knowledge acquisition to pathophysiological reasoning. LLMs currently demonstrate high proficiency in clinical pattern recognition and illness script matching, decreasing the utility of curricula focused primarily on pattern memorization (35). Prioritizing mechanistic understanding provides trainees with the foundational knowledge necessary to evaluate the physiological plausibility of AI-generated differential diagnoses and identify algorithmic errors (11). In the context of dual-process theory, prioritizing this mechanistic understanding strengthens students' analytical reasoning (System 2). This rigorous analytical foundation is essential to safely cross-check and critically evaluate the rapid, pattern-recognition outputs (akin to System 1) generated by AI tools.

To effectively cultivate these foundational competencies, UME programs are advised to move beyond traditional didactic lectures and integrate robust active learning strategies. Pedagogical approaches such as PBL and CBL could be adapted to include AI-generated distractors—for example, presenting students with a clinical case alongside an AI-generated differential that contains plausible but physiologically inconsistent options, compelling students to actively verify and critique the AI output against their mechanistic knowledge. Furthermore, concept mapping can be utilized to help students visually diagram complex pathophysiological mechanisms (36), ensuring they internalize disease processes rather than merely memorizing algorithmic outputs. These strategies directly address the never-skilling risk by ensuring that students engage in the cognitive struggle necessary to build durable illness scripts before they are permitted to rely on AI assistance in clinical settings.

Additionally, UME curricula are encouraged to incorporate data literacy and algorithmic bias education. Training in biostatistics and data science enables students to understand the curation, processing, and application of clinical data sets by machine learning algorithms (37). Instruction on algorithmic bias addresses documented health equity issues, as clinical algorithms can perpetuate racial and socioeconomic disparities in healthcare delivery (38, 39). Early integration of these competencies trains students to assess AI tools for equitable outcomes rather than accepting them as strictly neutral computational systems. For assessment, UME programs may consider supplementing traditional MCQs with oral case presentations and triple-jump exercises that require students to articulate their reasoning process aloud, making it difficult for AI assistance to substitute for genuine understanding.

### 4.2 Graduate medical education: human-AI collaborative decision-making

To counter these risks, GME needs to train residents in human-AI collaborative decision-making alongside independent diagnostic skills. The well-documented phenomenon of automation bias—whereby clinicians disproportionately defer to machine-generated recommendations even when they are incorrect—underscores the urgency of this training (18). This calls for instruction in adaptive AI engagement, utilizing frameworks such as the Best Available Human (BAH) principle. Under this framework, residents evaluate whether to utilize independent clinical judgment or algorithmic augmentation based on task risk and model validation status (40, 41). Clinical prompt engineering is a practical component of this training. Residents would benefit from formal instruction on constructing specific, context-rich queries and applying chain-of-thought prompting to improve LLM output accuracy and decrease confirmatory bias in model outputs (41, 42).

Furthermore, GME programs are advised to implement error correction and risk management protocols for AI use in clinical environments. The probabilistic nature of AI outputs adds a variable to standard clinical uncertainty (43). Supervision models, such as the DEFT-AI framework, provide structured oversight by requiring trainees to state their independent diagnostic reasoning before reviewing AI recommendations (41). This approach supports a “verify and trust” methodology, maintaining resident clinical autonomy while utilizing human-in-the-loop (HITL) oversight to manage diagnostic uncertainty and reduce automation bias (44).

Crucially, the cultivation of advanced clinical reasoning in the AI era requires deliberate practice. Drawing upon the theoretical framework of Schmidt and Mamede regarding deliberate reflection (45, 46), GME programs would benefit from incorporating structured clinical reasoning exercises. For example, residents can be tasked with analyzing complex case scenarios—first generating their own differential diagnoses independently, then reviewing an AI-generated differential, and finally composing a reflective written comparison that explicitly articulates where their reasoning converged with or diverged from the AI's output. This exercise not only reinforces independent reasoning but also develops the critical appraisal skills necessary to detect and reject incorrect AI recommendations, directly countering automation bias. For assessment, GME programs could consider supervised AI-use assessments integrated into clinical rotations, where supervisors directly observe how residents interrogate, verify, and integrate AI outputs into patient care decisions.

### 4.3 Continuing medical education: lifelong adaptation and unlearning

CME needs to expand its focus from periodic clinical knowledge updates to include continuous AI tool adaptation. While deskilling has been discussed in Section 3.1 primarily in the context of trainees, practicing clinicians are equally susceptible—particularly as AI tools become more integrated into daily workflows, potentially eroding diagnostic skills that were once maintained through frequent independent use. With LLMs serving as accessible medical knowledge repositories, lifelong learning frameworks are encouraged to emphasize the critical appraisal of AI outputs and the integration of AI-driven personalized, spaced-repetition learning (47, 48).

An additional component of CME involves the process of “unlearning,” defined in de-implementation science as the conscious alteration of knowledge, beliefs, and behaviors associated with obsolete practices (49). Operationalizing this involves a structured three-step de-implementation framework: (1) identifying the obsolete heuristic (e.g., relying solely on memory for complex drug interactions, or defaulting to a familiar diagnosis without considering less common alternatives), (2) substituting it with a verified AI-assisted protocol, and (3) reinforcing the new workflow through workplace evaluation. To illustrate: in the context of chronic pain management, a clinician might unlearn the heuristic of mentally retrieving a limited repertoire of analgesic options, and instead adopt a structured practice of querying an AI tool for a comprehensive, evidence-based list of alternatives—then critically appraising the AI's suggestions against the patient's specific comorbidities and contraindications. The integration of AI prompts a reassessment of certain cognitive heuristics, such as the availability heuristic and confirmation bias (50). Practicing clinicians are encouraged to identify reliance on these heuristics and substitute them with structured AI verification protocols (51, 52). To facilitate this, CME programs need to address cognitive habituation and professional resistance associated with adopting collaborative diagnostic models, thereby supporting the development of adaptive clinical expertise rather than static knowledge retention (49, 53). For assessment, CME portfolios could include documentation of AI-assisted clinical decisions with accompanying reflective justification, allowing clinicians and their supervisors to track how AI outputs were verified and integrated.

However, while structured “unlearning” of non-AI processes is a promising conceptual framework, further high-quality empirical research is urgently required to evaluate its effectiveness and safety in clinical practice before widespread implementation.

## 5 The re-emphasis on humanness and relational competencies

### 5.1 Humanness in clinical practice

Recent empirical evaluations indicate that AI-generated responses frequently score higher on text-based empathy metrics than physician-generated responses (54). However, experimental behavioral data demonstrate that the perceived therapeutic efficacy of an empathetic response is contingent upon its human origin. Identical text responses are rated as significantly more supportive when attributed to a human rather than an algorithm (55). This indicates that effective clinical empathy relies on moral subjectivity, phenomenal consciousness, and embodied experience—attributes absent in computational systems (56). Consequently, the literature distinguishes between diagnostic reasoning and management reasoning. While AI demonstrates high proficiency in diagnostic categorization based on pattern recognition, management reasoning—defined as the integration of patient values, the navigation of system constraints, and the establishment of a therapeutic alliance—remains a core human competency (35, 57).

### 5.2 Curricular shift toward relational competencies

The automation of cognitive-diagnostic tasks necessitates a realignment of medical curricula to prioritize relational and ethical competencies (6). This restructuring involves emphasizing the bedside clinical encounter, positioning the physical examination not solely as a data-gathering procedure, but as an application of therapeutic touch and clinical presence (58, 59). Furthermore, medical education is encouraged to longitudinally integrate methodologies such as narrative medicine, which demonstrates measurable efficacy in improving holistic patient connection, reflective observation, and empathic communication (60). Training in ethical decision-making needs to also adapt to AI-mediated care environments. Emerging frameworks, such as the Digital-Age Clinical AI Ethics Competence (DCEC) model, provide structured approaches for navigating algorithmic accountability, dataset bias, and epistemic opacity (61). Concurrently, instruction in shared decision-making needs to train clinicians to contextualize probabilistic AI outputs into patient-centered management plans. Crucially, the assessment of these humanistic competencies needs to evolve. Future evaluations could explore incorporating AI-integrated Objective Structured Clinical Examinations (OSCEs), where trainees interact simultaneously with a standardized patient and a clinical AI tool. Such assessments would evaluate the trainee's ability to maintain empathetic eye contact, navigate AI-generated diagnostic suggestions with the patient, and manage algorithmic uncertainty without eroding the therapeutic alliance. For instance, a specific assessment rubric for these OSCEs could include weighted criteria evaluating whether the trainee explicitly communicates AI-generated diagnostic uncertainty to the patient, maintains empathetic eye contact rather than focusing solely on the screen, and successfully negotiates a shared management plan.

### 5.3 Functional division of labor in clinical workflows

Current educational and clinical discourse proposes a functional division of labor. This operational paradigm is characterized by AI managing the electronic health record (EHR)—effectively “treating the chart”—while the clinician focuses on direct, relational patient care (62). It is important to note that this division is a conceptual framework rather than a strict operational rule—the clinician retains ultimate accountability for all AI-generated outputs and the final clinical decisions, and the boundary between documentation, diagnostic reasoning, and patient interaction is frequently inseparable in real clinical practice. For trainees, the act of clinical documentation is itself a vital cognitive process that facilitates diagnostic reflection. Therefore, this proposed division of labor should be framed explicitly as a pedagogical heuristic—a conceptual guide for curriculum design—rather than a strict operational reality.

**Figure 3:**
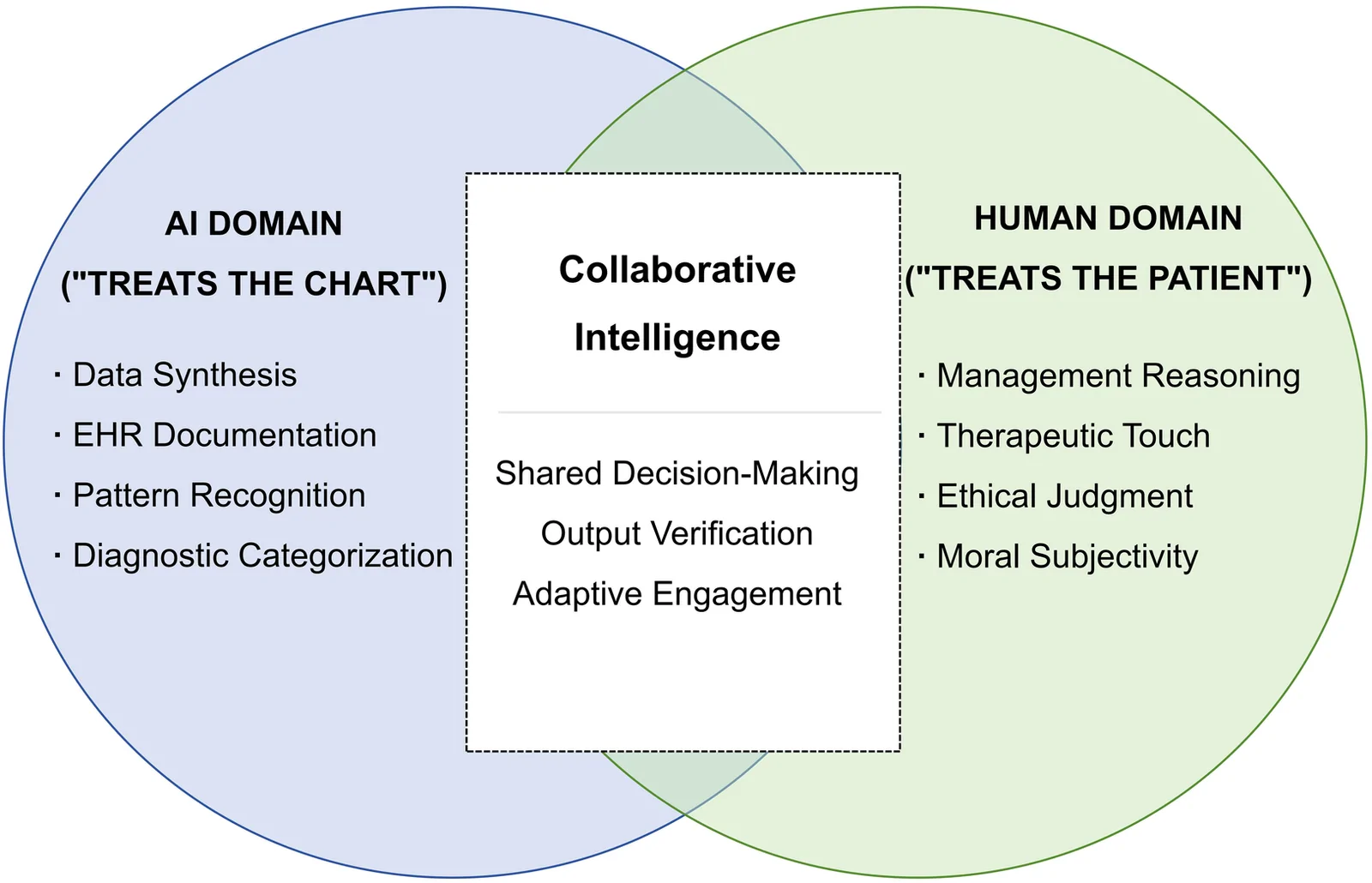
The functional division of labor: the “Augmented Clinician” model. A Venn diagram illustrating the optimal triadic clinical workflow in the era of generative AI. The model delineates the specific responsibilities of the AI domain (focused on data synthesis and chart management) and the irreplaceable human domain (focused on management reasoning, ethics, and therapeutic connection). The intersection represents “Collaborative Intelligence,” where clinicians engage in shared decision-making and rigorous output verification to integrate algorithmic data with human-like patient care.

This operational paradigm redefines the triadic clinical workflow, distinguishing between algorithmic data processing and humanistic management reasoning (Figure 3). Ambient AI documentation systems demonstrate measurable efficacy in reducing electronic health record task load and clinician burnout, theoretically reallocating time for direct patient interaction (63, 64). However, integrating this model into medical training introduces specific vulnerabilities. The delegation of clinical documentation to AI systems removes a primary cognitive mechanism through which trainees synthesize clinical data and develop professional reasoning (65). Furthermore, without strict institutional governance, health systems may reallocate the time saved by AI toward increased patient throughput rather than enhanced relational care (64). Addressing these risks highlights the need for medical education to train physicians in task-based adaptive practices, ensuring that clinical oversight is maintained and that technology is utilized to augment, rather than displace, the fundamental elements of patient care (41, 66).

### 5.4 Implementation barriers and the efficiency paradox

While the transition toward an AI-augmented, humanistic curriculum is conceptually robust, significant implementation barriers exist. Current literature suggests that a deficit in AI literacy among some medical school faculty may hinder the effective teaching and assessment of algorithmic stewardship (67, 68). Beyond literacy deficits, institutions face significant logistical and financial barriers. Implementing the aforementioned strategies—such as reflective writing, complex case scenarios, and structured clinical reasoning exercises—imposes a substantial assessment burden. Providing high-quality, individualized feedback is particularly challenging for medical schools with large student cohorts, severely straining faculty workload and time constraints. Additionally, the financial cost of subscribing to secure, HIPAA-compliant advanced AI platforms adds considerable strain to institutional budgets, particularly for resource-limited institutions (69). This financial barrier is compounded by the need for ongoing technical support, regular model updates, and faculty training—costs that are often underestimated in initial budget planning (66). To mitigate these barriers, institutions might need to explore peer-to-peer assessment models, utilize standardized grading rubrics for reflective writing, and implement a “train-the-trainer” approach to distribute faculty workload more efficiently.

Furthermore, implementation challenges are exacerbated by global inequities. Many institutions, particularly in low- and middle-income countries (LMICs), lack access to commercial AI platforms, faculty development programs, or robust digital infrastructure. Addressing these global disparities is vital to ensure the international relevance and equitable implementation of AI-integrated medical education frameworks. Most critically, an “efficiency paradox” looms over the humanistic resurgence. While ambient AI scribes theoretically save time for relational care, there is a risk that healthcare administrators will exploit these efficiencies to increase patient throughput—mandating shorter clinical encounters rather than deeper therapeutic connections. To prevent the erosion of humanism by efficiency logic, the integration of AI needs to be accompanied by stringent institutional policies that explicitly protect clinician-patient interaction time.

## 6 Conclusion

The integration of generative AI into medical education represents a paradigm shift that is already reshaping how clinical knowledge is acquired, assessed, and applied. As this review has argued, while AI offers unprecedented opportunities for personalized learning and cognitive offloading of administrative tasks, it simultaneously introduces three specific cognitive vulnerabilities that medical educators need to proactively address: never-skilling among junior trainees who are exposed to AI before establishing foundational mental models; deskilling among advanced learners whose independent diagnostic abilities may deteriorate through sustained algorithmic dependence; and automation bias, whereby clinicians uncritically accept machine-generated recommendations, particularly when independent verification is challenging.

To counter these vulnerabilities, we have proposed a phase-specific structural realignment across the medical education continuum. At the UME, curricula are encouraged to prioritize pathophysiological reasoning, data literacy, and algorithmic bias education—with active learning strategies such as PBL/CBL incorporating AI-generated distractors and concept mapping—to ensure that students build durable illness scripts before engaging with AI tools. We acknowledge that many of the educational strategies we recommend—such as case-based learning, concept mapping, and reflective exercises—have long been promoted in competency-based medical education frameworks. What is distinct in the current context is the urgency and specificity with which generative AI now demands their systematic implementation, as well as the novel need to explicitly train students in AI-output verification within these established pedagogical structures.

At the GME, training would benefit from focusing on human-AI collaborative decision-making, structured prompt engineering, and deliberate reflection exercises that require residents to compare their independent reasoning against AI outputs, thereby maintaining cognitive autonomy while leveraging algorithmic support. At the CME, lifelong learning frameworks need to extend beyond knowledge updates to include structured “unlearning” of obsolete heuristics, supported by the three-step de-implementation process of identifying, substituting, and reinforcing new AI-verified workflows.

Running throughout these phase-specific recommendations is a unifying theme: as AI becomes increasingly proficient at data synthesis, pattern recognition, and documentation tasks, the physician's irreplaceable value lies in management reasoning, ethical judgment, and relational care—the humanness that no algorithm can replicate. We have therefore advocated for a functional division of labor in clinical workflows, where AI “treats the chart” and clinicians focus on the therapeutic alliance, while acknowledging the implementation barriers—faculty AI literacy deficits, institutional costs, assessment burden, and the “efficiency paradox”—that need to be addressed through institutional governance and policy safeguards.

Limitations of this review. As a narrative and critical review, this article does not constitute a systematic quantitative synthesis of the literature. Our proposed framework and recommendations are conceptually derived from the available evidence but require empirical validation through prospective, longitudinal studies that measure the long-term impact of AI-integrated curricula on clinical reasoning outcomes and, ultimately, patient safety (Kirkpatrick Level 4). Furthermore, validated psychometric instruments to assess human-AI collaborative competence in clinical settings remain underdeveloped and represent a critical priority. Future empirical research should specifically prioritize: (1) longitudinal cohort studies evaluating clinical reasoning development over time; (2) randomized educational interventions comparing AI-assisted vs. traditional curricula; (3) the psychometric validation of AI competency frameworks; and (4) downstream studies examining actual patient safety outcomes following AI-integrated education.

Ultimately, the goal of medical education in the AI era is not to compete with algorithmic efficiency, but to cultivate the “augmented clinician”—a physician who possesses high AI literacy, exercises rigorous independent judgment, and remains deeply grounded in the humanistic foundations of healing. Ensuring that technological integration enhances, rather than displaces, the relational core of patient care must remain the guiding principle of medical education reform.
